# Annus Medicus, 1895

**Published:** 1896-01-04

**Authors:** 


The Hospital

AN INSTITUTIONAL JOURNAL OF

The Medical Sciences and Hospital Administration,

WITH

AN EXTRA NURSING SUPPLEMENT.

VOL. XIX.—No. 484.] [JAN. 4, 1896.

### ANNUS MEDICUS, 1895.

FAR FROM THE GOAL.

THAT medicine is rising higher in public estimation year by year we have the best of reasons for believing. The Marquis of Salisbury, Mr. Gladstone, and Mr. Balfour have all assured us of the fact. That it has reached the elevation and influence which it is destined to attain, and which, for the world's sake, we may hope it will attain without undue delay, cannot yet be affirmed. It is manifest that, although men of "light and leading" have begun to value us and our science with reasonable accuracy, many of the public still look upon us as a class of more or less superior nurses, whose business is solely at the bedside or by the valetudinarian's chair, and whose opinions are of no moment whatever at a critical juncture in State affairs. This should not be so. The highest interests of the communities of which nations are constituted demand that physiological and medical science shall not merely take a lead but shall attempt nothing less than the gigantic task of recasting civilisation in a scientific mould. Such is the call which civilisation is making upon physiological and medical science at the present moment.

SERUM THERAPY.

The year 1895 has been most interesting to the student of medical progress. Whatever may or may not be said of medical science, everybody must admit that it is "very much alive." The most "burning" question of the year has, perhaps, been that of "serum therapy." As to what serum therapy will or will not ultimately accomplish for medicine, no man of philosophic mind will at present speak with decision. The subject was very fully and competently discussed at the meetings of the British Medical Association held in London in July and August. The majority of those who were entitled, by experience and general medical capacity, to speak, were decidedly of opinion that so far as diphtheria is concerned, the serum anti-toxin employed in its treatment is a powerful agent for good. It appeared from their accounts that the disease processes were favourably and very speedily modified by its use; convalescence was established with promptitude; and recovery was swift and sure. It was averred and accepted at the meetings of the Association that the mortality from diphtheria was reduced by 25 per cent. wherever the anti-toxin was skilfully employed. Adverse critics, who spoke of injuries to kidney structure, and of mischief wrought in other parts of the body, did not substantiate their criticisms by competent evidence. It may, therefore, be stated that, for the present, "serum therapy" holds the field as an effective treatment for diphtheria in the estimation of those who are generally held to be worthy of the confidence of the profession. On the general subject of anti-toxins, it is worth while to reproduce a HOSPITAL summary of an article by Dr. Klein, which appeared in "Science Progress" for March: "Some observers think that toxin is converted into anti-toxin by the action upon it of the living cells of an animal. Others, and to this view Dr. Klein inclines, believe that anti-toxin is a direct product of the living cells themselves, produced under the stimulus or irritation of toxin. There is an important practical difference between these two views. If anti-toxin is merely altered toxin, we may hope to prepare it artificially in our laboratories without the agency of living animals. But if, on the other hand, it is a product of some change in the cells themselves of the organism, a change brought about by the action of toxin on those cells, then it seems certain that we shall always have to prepare anti-toxin by means of the action of toxin upon living animals, and in no other way. Dr. Klein adds, as a last important fact, that the substance itself of bacterial bodies is anti-toxic; and that immunity against infective disease is acquired not only through the agency of anti-toxin evolved from the cells of an organism under stimulus, but from the *débris* of the disintegrated specific organisms themselves." The therapeutical significance of all this is obvious. It is obvious also that the whole question of serum therapy is charged with difficulties; and that much more scientific work, both in the laboratory and at the bedside, will have to be carried out by competent brains and hands before the final word can be said on the subject. In the meantime it is desirable that clinical experience should not be derived only from hospitals. Every member of the profession who happens to be blessed with an inquiring mind can assist in the investigation.

THE PROGRESS OF SURGERY.

At the Congress of Surgery, held at Berlin in April, Professor von Bergmann discussed the question of "Recent Progress in Cerebral Surgery," and pointed out that the enormous strides which had been made in this department of medicine were due to two principal factors—namely: First, the localisation of cerebral function; and, secondly, the perfection of modern antiseptic methods of treatmeot. Professor Bergmann had nothing specially new to record; and it may, therefore, be worth while to devote a sentence or two to the latter of his two factors of modern surgical progress. To antiseptics is principally due the great advance of surgery in recent times. Murphy has opened the abdominal cavity 194 times for appendicitis, with a mortality of 9·6 per cent., and now holds the opinion that every case of appendicitis, promising or unpromising, should be operated upon. But let us now turn for a moment to another aspect of "surgical progress": An eminent bacteriologist affirmed during the year 1895 that there is hardly a surgeon in London who has passed the age of fifty-five who has "any complete comprehension of the antiseptic system in its detailed application to the art of modern surgery." What then does "surgical progress" mean? Can that be properly called "surgical progress" which is merely the progress of an isolated individual here and there in surgery? It is now nearly twenty years since Sir Joseph Lister made almost the completest practical demonstration which has ever been known in ancient or modern medicine. That demonstration has been accepted by the whole scientific world for close upon twenty years, and yet we are told that in actual surgical practice in London, the very centre of the British surgical world, antisepticism, though thus convincingly demonstrated, and redemonstrated tens of thousands of times since in every hospital of the civilised world, is not even comprehended, save by an insignificant fraction, among all the surgeons who have reached middle life. Concerning which, the best that can be said is that we most fervently hope it is not true.

As if to add emphasis to our contention that the greatest fact of surgical progress in modern times is the principle and method of antisepticism, the Royal Society selected the year 1895 for the appointment of Sir Joseph Lister as its president, in succession to one of the most distinguished presidents, Lord Kelvin, who has ever lent lustre to that great home of science. Sir Joseph Lister will reign over the British kingdom of science with the heartfelt approval of every scientific man among us, and with the general endorsement of the whole scientific world. If that be indeed so, may we not expect that every man who is called to do surgical work will feel that his scientific conscience can by no means be fully satisfied until he has mastered in principle and detail, and carried into every department of his life and work, the great discovery which it has been the distinguishing honour of our country in the person of Sir Joseph Lister to make available for the relief and physical salvation of suffering mankind?

### Medicine.—The Royal Commission on Tuberculosis.

About five years ago a Royal Commission was appointed to investigate the nature and extent of human and animal tuberculosis, and to report. The Commission met at intervals until the early part of the year, and has since issued a report. As every man of bacteriological knowledge expected, the Commissioners have published the conclusion that tuberculosis in warm-blooded animals is identical with tuberculosis in man. It is a microbic, an infectious, and may become a deadly disorder wherever it is found. One practical conclusion from this is that the flesh of tuberculous animals is a dangerous poison. The experiments of Dr. Sims Woodhead and Dr. Sidney Martin on the point are quite conclusive. In this connection we may remind readers that experiments are still being made, more especially on the Continent, to produce a serum which shall, when inoculated into healthy subjects, prevent tuberculosis altogether, as vaccination prevents small-pox, or shall cure the disease where already established, at any rate, in the earlier stages of its progress. The Italian professor, Maragliano, has sprung into some notoriety in this department of scientific activity. He has not only produced, but also experimented, and as he says, successfully, with an anti-tuberculous serum. Unfortunately, like Koch, he has kept his method of producing his serum a profound secret. The interest of straightforward men of science is largely held back by this procedure. For ourselves, we cannot but reprobate all such unscientific and vulgar methods, by whomsoever they may be initiated and carried out. True science hangs down her head in shame whenever such things are done.

### Genius, Insanity, and Degeneracy.

England has always stood for sanity in her men of genius. She points to her Shakespeare, her Milton, and her Tennyson, her Locke and her Mill, her Butler and her Hooker, her Darwin, her Huxley, her Kelvin and her Lister, her Freeman, her Seeley, and her Green. To her, therefore, Lombroso, Max Nordau, and others of their school, appear to be themselves lacking in the very highest sanity when they proclaim that there is any necessary connection, any true kinship, between genius and insanity. The science of the subject may be summed up in a few words. The mind of man works by means of a physical medium—the brain. That physical medium, like anything else that is at once physical and vital, is liable to injury, and to the effects of wear and tear, as also to peculiarities of structure entailed by heredity. The brain of the genius is as much liable to injury, and to the effects of wear and tear, as also to the peculiarities of structure entailed by heredity, as the mind of every other order of human beings; no more, no less. As of old, not only is the highest genius compatible with the highest sanity, but the very height and perfection of all genius is sanity in its highest and broadest perfection. In a word, sanity is genius; genius is sanity.

The question of pure and simple "degeneracy," raised so opportunely by Max Nordau, is of a different order. That a "wave of degeneracy" has been passing over Europe we do not deny; that many, even of European men of genius, during the past half century, have been more or less insane we may readily admit. That, however, in no wise touches our fundamental contention that genius has no necessary connection with insanity. In a degenerate and degenerating world, a world where all men are at a low ebb in point of sanity, the genius of the time will partake of the insane characteristics of the time. On the other hand, in a period of outdoor activities, healthy occupations, reasonable contentment, religious hopes, and "plain living and high thinking," the mass of mankind will be eminently sane, and the genius of the time will be sane in its own measure just as the great mass is sane. We have heard too much about genius and insanity. Let us hope that some man of science, whose time is not better employed, will now proceed to tell us something of "genius and sanity."

### Hospital Progress.

In a very wide sense medical progress is hospital progress, and hospital progress is medical progress. But there is an aspect of hospital activity which is financial and administrative rather than medical, and which is of present practical importance. The Hospital Sunday Fund is a kind of hospital barometer, which indicates fairly well the state of the mental atmosphere of the public in relation to hospitals. This year, very fortunately, the barometer has indicated an unusual measure of sunshine and fine weather. The Sunday Fund has leaped up from a little more than forty to something over sixty thousand pounds. We may, we think, be permitted to congratulate the hospitals a great deal, and ourselves a little, on this achievement. It has been acknowledged, both by the Sunday Fund itself, and by the general press, that The Hospital's special Sunday number made a unique attempt to present the great facts and successes of hospital life and work in a clear and convincing form to the philanthropic public, and achieved a unique success. More than this we do not desire to say. We rejoice with the Fund and the hospitals in their new prosperity, and express the fervent hope that it may be continued and increased fourfold. Whilst on this subject we may remind readers that an attempt was made during the year in connection with the Great Northern Central Hospital to modify to some extent in favour of the general practitioner of medicine the very hard and fast lines of hospital administration which mark the hospital life of the metropolis. Coupled with this was an effort to reconsider a modified pay system, also made with a view to the diminution of the competition which is believed to exist between hospitals and family physicians. Those efforts, we regret to say, were frustrated by the quite remarkable timidity of Mr. C. T. Murdoch, M.P., the chairman of the Great Northern Central Hospital's Committee. Of course the subject will come up again, and most probably at the same hospital. There are a few men of business on its committee who are by this time heartily ashamed of the futilities of the last annual meeting. That hospitals will see the general drift of modern progress, and will "change with the changing times." is as certain as that they will continue their prosperous activity. Life means change. If hospital committees resist all change, even change for the better, they do but sound the knell of their own dissolution.

### The Great Dead.

Some men of the first eminence have dropped from the ranks of medicine and science during the year; of these we will but mention three.

Pasteur was to us a foreigner, and courtesy therefore gives to him the place of honour in a British paper. His achievements we need not recall; they were truly scientific, they were great, they were helpful to humanity at large. Pasteur himself was the kind of man whom all men love. Great in his work, he was modest in his life. He commanded the homage of mankind by his achievements; but he never claimed it. He was above all things a true man. He, was, in the commonly accepted sense of the word, a good man—a man who did not separate himself from the great mass of mankind in his principles, his hopes, and his beliefs. He lived and died in the Catholic faith. May his soul rest in peace.

Thomas Henry Huxley was our own man of science. He too has died, and died whilst yet scarcely old. Typical he was, and representative. English science has long been agnostically inclined. It has aimed at being very true, very genuine—at sticking to facts, and recording only what it could prove. This is part of the British character, which hates pretence and make-believe. Huxley was of the very essence of the sublimated Englishman, and, therefore, he sometimes went wrong. But when he went wrong, he went wrong like a man; when he fought, he fought like a man; when he recanted, as he did sometimes, he did it like a man. He was a man of science, but also a man of affairs. He found in some of the highest life a good deal of outside show, which had ceased to represent any inner significance. He found this, as he thought, among religious people in especial. Like a true man, he charged make-believe in religion full in front, exactly as he would have charged make-believe in science or in politics. Therein we cannot but think he rendered good service, even to religion itself. Be that as it may, the man of science recognised in Huxley a potent force making, on the whole, for truth in science and for righteousness in action; and therefore science acclaims Huxley not only a great man, but a good. His name will be remembered as long as British science plays a leading part in the world.

Another name we record, the name of Thomas Syer Bristowe, M.D., F.R.S., man of science and accomplished physician. Dr. Bristowe was younger than either Huxley or Pasteur when he died. As learned and as truly scientific as either, he had not achieved the world-wide reputation which made them so universally mourned. But this much may be said with perfect truth, that many a man with but a fraction of the learning and science, and with none at all of the high and noble character, of Dr. Bristowe, has been held to be great in every civilised land. Dr. Bristowe was truly great, both in himself and in his work. He was great to the few who knew him. If the world had ever known him, as it would have done if he had survived but a few years longer, it too would have made a recognition of his greatness which would have surprised no one more than himself. Most truly worthy was Dr. Bristowe of all the deep affection and honour which those who knew him always felt for him.

Many other men of science and members of the medical profession have died during the year, whose names and honours we should wish to recall did space permit. Their science has helped to mitigate the sufferings and sorrows of the world, their personal worth has tended to sweeten and ennoble life.

### The New Year.

The past is gone. To the true man of science the future is ever the most inspiring. He considers that what is done is nothing in the presence of the vast achievements which yet await his strenuous efforts. Of no science can this be more truly said than of medicine. Her great aim, if that were possible, would be the complete abolition of all sickness and suffering. Compared with this the work already accomplished is but as an alphabet of a universal language. Can any aim be greater? It is for all of us who claim to be men of science, as well as physicians and surgeons, to see and recognise the vast extent of the work which lies before us; and it is for the great world also to see the gigantic nature of the task which destiny has laid upon us, and to give us in unstinted measure its recognition, its sympathy, and its help.

